# The Impact of Urban Pollution on Plasmid-Mediated Resistance Acquisition in Enterobacteria from a Tropical River

**DOI:** 10.3390/antibiotics13111089

**Published:** 2024-11-14

**Authors:** Bradd Mendoza-Guido, Kenia Barrantes, César Rodríguez, Keilor Rojas-Jimenez, Maria Arias-Andres

**Affiliations:** 1 Instituto de Investigaciones en Salud, Universidad de Costa Rica, San José P.O. Box 11501-2060, Costa Rica; bradd.mendoza@ucr.ac.cr (B.M.-G.); kenia.barrantes@ucr.ac.cr (K.B.); 2 Programa de Doctorado en Ciencias Naturales para el Desarrollo, Universidad Estatal a Distancia, San José P.O. Box 474-2050, Costa Rica; 3 Centro de Investigación en Enfermedades Tropicales, Facultad de Microbiología, Universidad de Costa Rica, San José P.O. Box 11501-2060, Costa Rica; cesar.rodriguezsanchez@ucr.ac.cr; 4 Escuela de Biología, Universidad de Costa Rica, San José P.O. Box 11501-2060, Costa Rica; 5 Instituto Regional de Estudios en Sustancias Tóxicas, Universidad Nacional de Costa Rica, Heredia P.O. Box 86-3000, Costa Rica

**Keywords:** Enterobacteria, Costa Rica, integrons, plasmid evolution, antibiotic resistance, horizontal gene transfer, *Escherichia coli*, *Klebsiella*, IncH plasmids, beta-lactamase

## Abstract

**Background:** The exposure of environmental bacteria to contaminants in aquatic ecosystems accelerates the dissemination of antibiotic-resistance genes (ARGs) through horizontal gene transfer (HGT). **Methods:** In this study, we sampled three locations along a contamination gradient of a polluted river, focusing on isolating Enterobacteria from the surface waters to investigate the relationship between urban pollution and antibiotic resistance. The genomes of 15 isolates (5 per site) were sequenced to identify plasmid-borne ARGs and their association with resistance phenotypes. **Results:** Isolates from the site with the highest contamination (Site 3) showeda larger number of ARGs, plasmids, and resistance phenotypes. Notably, one of the isolates analyzed, *E. coli* A231-12, exhibited phenotypic resistance to seven antibiotics, presumably conferred by a single plasmid carrying 12 ARGs. Comparative analysis of this plasmid revealed its close evolutionary relationship with another IncH plasmid hosted by *Salmonella enterica*, underscoring its high ARG burden in the aquatic environment. Other plasmids identified in our isolates carried *sul* and *dfrA* genes, conferring resistance to trimethoprim/sulfamethoxazole, a commonly prescribed antibiotic combination in clinical settings. **Conclusions:** These results highlight the critical need to expand research on the link between pollution and plasmid-mediated antimicrobial resistance in aquatic ecosystems, which can act as reservoirs of ARGs.

## 1. Introduction

The contamination of water resources with organic compounds of anthropogenic origin has exceeded the capacity to monitor and evaluate their harmful effects on environmental health [1]. The presence of chemical contaminants, including emerging pollutants (EPs) such as microplastics, pharmaceuticals, pesticides, and heavy metals, can elevate the abundance of antibiotic-resistance genes (ARGs) in aquatic environments, even without antibiotics. Experimental studies have shown that non-antibiotic compounds can impact horizontal gene transfer (HGT) dynamics [2,3], increasing plasmid transfer rates across diverse bacterial taxa. These contaminants exert selective pressures that not only promote ARG proliferation but also generate mutations in antibiotic target genes. For instance, they can induce oxidative stress in bacteria, triggering DNA damage and activating repair pathways like the SOS response, facilitating the acquisition of new mutations. Concurrently, oxidative stress has been observed to alter cell membrane permeability and to up-regulate genes associated with conjugation, thus enhancing both plasmid conjugation rates and transformation frequencies [2,3,4].

Wastewater (with or without treatment) contains complex mixtures of chemical residues used by the human population (e.g., pesticides, antibiotics, and other pharmaceutically active compounds) that are accumulated and released into the environment. Urban growth increases the sources of these pollutants in river basins significantly, and urban populations will only continue to increase [5]. Further, in economies with scarce wastewater management, resistance-selecting chemicals can be detected at higher concentrations in surface waters [6,7]. This might cause selective pressure over the exposed microbial communities, leading to the dissemination of ARGs in the ecosystem.

Bacterial species commonly found in aquatic environments, such as *Escherichia coli*, *Acinetobacter baumannii*, and *Pseudomonas* spp., are frequently associated with plasmids that carry antibiotic-resistance genes (ARGs). In aquatic settings, biofilms are crucial in enhancing plasmid retention and transfer. Bacteria in biofilms, due to their close physical proximity, experience higher rates of conjugation, allowing plasmids to be transmitted more efficiently compared with planktonic (free-floating) bacterial populations [8,9]. Due to that, biofilms can serve as reservoirs for plasmids with ARGs, protecting them from being lost in the absence of antibiotics, but they also help to preserve genes required for plasmid transfer. This contrasts with planktonic populations, where plasmids are often lost due to selective sweeps or genetic deletions that eliminate transfer regions [8]. The spatial structure and local competition within biofilms enable bacteria to maintain these plasmids longer, contributing to the persistence of ARGs in aquatic systems, even without antibiotics. This dynamic highlights the biofilm’s critical role in sustaining plasmid-mediated antibiotic resistance in bacterial communities.

Although antibiotic resistance has emerged across various bacterial groups, bacteria from the family Enterobacteriaceae are of particular concern due to their critical role in the intestinal microbiota and significance in human and animal health. Certain species within this group exhibit high levels of antimicrobial resistance, raising substantial concerns regarding their potential impact on human health [10]. In this study, we aimed to characterize the diversity of plasmid-mediated ARGs in Enterobacteriaceae isolated from water column samples along a gradient in pollution and anthropogenic activities (urbanization). Samples were collected from three sites along the main course of the Virilla River, situated in the Western Central Valley of Costa Rica (Supplementary Figure S1). The Virilla River is part of the larger Grande de Tárcoles River Watershed, which flows into the Pacific Ocean. Urbanization and shifts in land use within this watershed have significantly increased pollution levels, mainlydue to untreated municipal sewage and industrial discharges [11,12]. These findings emphasize the potential role of urban pollution in driving the spread of antibiotic resistance in clinically important bacteria, highlighting the key role that plasmids play in this process.

## 2. Results

### 2.1. Genome Characterization and Taxonomic Identification

In Table 1, we present the characteristics of the assembled genomes and their taxonomic classification as determined using the Type Strain Genome Server (TYGS) [13]. Eight of the 15 genomes analyzed were identified as *Escherichia coli* based on TYGS, while 4 additional isolates were classified as *Klebsiella pneumoniae*, 1 as *Klebsiella variicola*, and another as *Klebsiella quasipneumoniae* (Supplementary Figure S2). TYGS could not identify the isolate A223-7 at the species level but associated its genome with the *Citrobacter* genus; however, the GTDB tool indicated a close relationship to *Citrobacter gillenii*, with a 98.96% average nucleotide identity (ANI).

**Figure 1:**
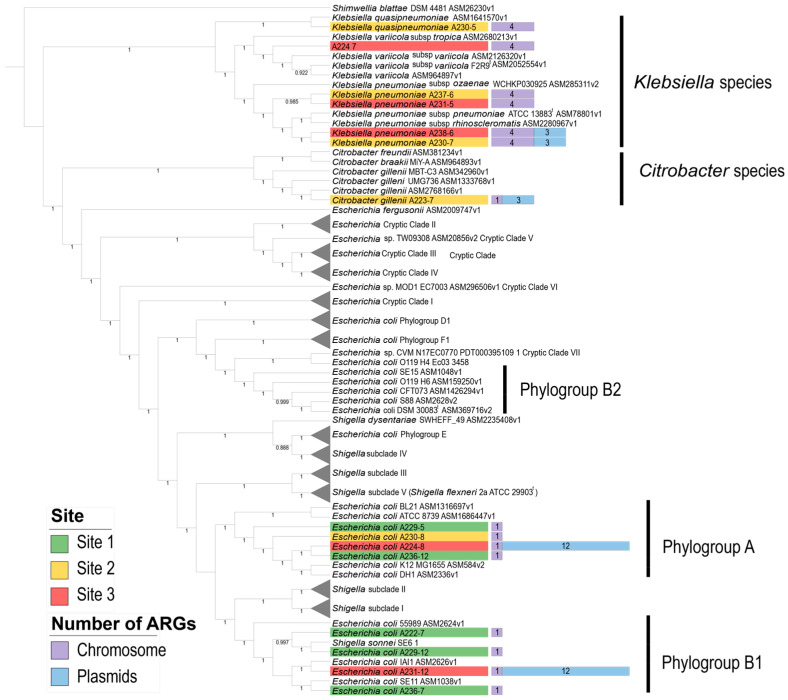
Phylogenomic tree representing the evolutionary relationships of the isolated bacteria and reference genomes. The tree was constructed using 908 single-copy core genes shared across all the genomes. Bootstrap values, based on 1000 resamplings, appear at the nodes. The height of the bars corresponds to the number of ARGs found in the chromosome (purple) and plasmids (blue), with the label colors indicating the sites where the bacteria were isolated. *Escherichia* and *Shigella* clades unrelated to the isolates were collapsed to reduce the number of labels. The *E. coli* phylogroup B2 is highlighted due to the inclusion of the type strain *E. coli* DSM 30083^t^. Branch lengths were omitted for improved visualization.

To further clarify the isolates’ taxonomic assignment and genomic relationships, we conducted a phylogenomic analysis using 908 single-copy core genes. We show in Figure 1 the phylogenomic relationships between the isolates and related genomes obtained from the NCBI database, including reference genomes for each species and representative strains for different *Escherichia coli* phylogroups and *Shigella* subclades. The NCBI assembly names of all the external genomes used in this phylogenomic analysis are listed in Supplementary Table S1.

As previously classified, six isolates were assigned to the genus *Klebsiella*: isolate A224-7 is related to *Klebsiella variicola*, isolate A230-5 to *Klebsiella quasipneumoniae*, and isolates A237-6, A238-6, A230-7, and A231-5 to *Klebsiella pneumoniae*. Additionally, isolate A223-7 was confirmed to be related to *Citrobacter gillenii*, consistent with the GTDB tool’s results. For the isolates classified as *E. coli*, we determined that they are grouped into two distinct phylogroups: phylogroup A, which includes isolates A224-8, A229-5, A230-8, and A236-12, and phylogroup B1, which contains isolates A236-7, A229-12, A222-7, and A231-12, along with the *Shigella sonnei* SE6-1 strain, which appears to have been misidentified as a *Shigella* strain [14].

### 2.2. Phenotypic Characterization of Antibiotic Resistance

**Figure 2:**
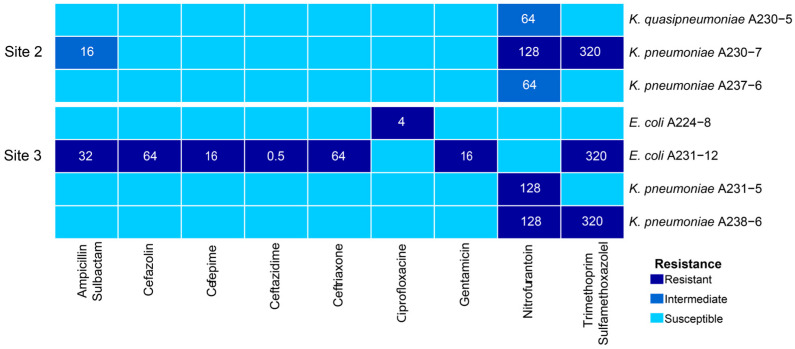
Antibiotic susceptibility testing of the evaluated isolates. Each box shows the minimum inhibitory concentration (MIC) obtained, and the colors represent the resistance phenotype: dark blue for resistant, blue for intermediate-resistant, and light blue for susceptible isolates. Resistance levels were assessed based on the MIC breakpoints for Enterobacterales as outlined in the 2020 CLSI guidelines [16].

Seven of the 15 evaluated isolates exhibited resistance to at least 1 of the 15 antibiotics evaluated (amikacin, gentamicin, ampicillin/sulbactam, ertapenem, meropenem, cephalothin, cefazolin, ceftazidime, ceftriaxone, cefepime, ciprofloxacin, norfloxacin, trimethoprim/sulfamethoxazole, fosfomycin, and nitrofurantoin) (Figure 2). None of the isolates from Site 1 (the least contaminated) displayed resistance to the tested antibiotics, whereas three isolates from Site 2 and four from Site 3 (the most contaminated) showed resistance to one or more antibiotics. Among the multidrug-resistant isolates (defined as resistance to three or more antibiotics) [15], *K. pneumoniae* A230-7 showed resistance to nitrofurantoin, trimethoprim/sulfamethoxazole, and an intermediate level of resistance to ampicillin/sulbactam. Meanwhile, *E. coli* A231-12 exhibited resistance to seven antibiotics—ampicillin/sulbactam, cefazolin, cefepime, ceftazidime, ceftriaxone, gentamicin, and trimethoprim/sulfamethoxazole—and was identified as an extended-spectrum beta-lactamase (ESBL) producer. Notably, five isolates presented resistance to nitrofurantoin, while three were resistant to trimethoprim/sulfamethoxazole.

### 2.3. Genotypic Characterization of Antibiotic Resistance

Across the 15 sequenced genomes, we identified 66 ARGs, 34 of which were unique (richness). Of these 66 ARGs, 33 (18 unique) were in bacterial chromosomes, while the remaining 33 ARGs (16 unique) were in plasmids. It was remarkable that no ARGs were detected in both the chromosome and plasmid contigs of the same isolate except for the *E. coli* A224-8 isolate, which presented a plasmid sequence integrated into the bacterial chromosome. However, given that this might be a sequencing artifact, the sequence was classified as a plasmid. We provide all information regarding the ARGs identified by the ABRicate v1.0.0 software using the NCBI database in Supplementary Table S2.

At Site 1, we determined an abundance and richness of ARGs of 5 and 3, respectively. In contrast, Site 2 presented 20 ARGs, 19 of which were unique, while Site 3 showed the highest diversity values with a total of 41 ARGs, 29 of which were unique. Additional statistic values are presented in Table 2. As the most contaminated site, Site 3 contained the largest number of ARGs, followed by Site 2, with Site 1 having the lowest diversity of ARGs. We identified statistically significant differences in ARG distribution across the sites (Kruskal–Wallis, χ^2^ = 9.95; *p* = 0.007). However, post hoc Dunn tests showed significance only between Sites 1 and 3 (Z = −3.130, *p* = 0.005) but not between Sites 1 and 2 (Z = −1.900; *p* = 0.086) or between Sites 2 and 3 (Z = −1.230; *p* = 0.219). The isolates with the highest richness of ARGs were *E. coli* A231-12 (Site 3) with 13, *E. coli* A224-8 (Site 3) with 10 (11 after sequencing with PacBio), and *K. pneumoniae* isolates A238-6 (Site 3) and A203-7 (Site 2), both with 7 ARGs.

**Figure 3:**
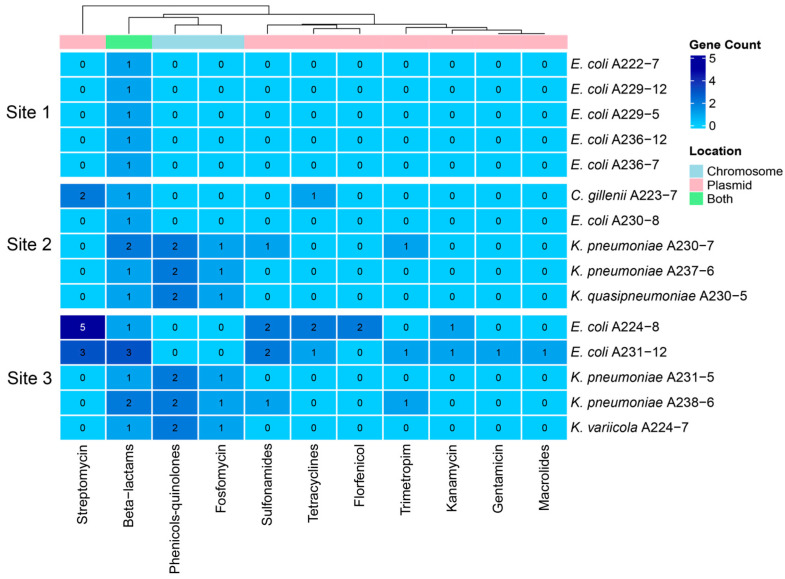
Potential phenotypes associated with ARGs identified in each genome analyzed. The heatmap color gradient represents the gene count of ARGs, while the column colors indicate the genomic location of the genes: light blue for chromosomal, pink for plasmid, and green for both chromosomal and plasmid locations.

Concerning the differences in plasmid- or chromosomal-encoded ARGs, we identified 15 chromosomal ARGs associated with extended-spectrum beta-lactamases (ESBL) and 12 chromosomal ARGs from the *oqx* family, which encode RND efflux pumps linked to resistance against phenicol, quinolones, and nitrofurantoin [17,18]. Notably, streptomycin-resistance genes (10 ARGs) were observed exclusively in plasmids, encoding phosphotransferases and nucleotidyl transferases that modify ribosomes and allow evasion toaminoglycoside antibiotics. Additionally, we identified plasmid-encoded ARGs conferring resistance to sulfonamides (six ARGs) and tetracyclines (four ARGs) among others, as listed in Figure 3.

When analyzing point-mutation-associated resistance, we determined that *E. coli* A224-8 (Site 3) was the only one exhibiting a mutation in the *gyrA* gene (S83L), which confers resistance to ciprofloxacin. Additionally, mutations in the *nfsA* and *nfsB* genes, along with potential ARGs conferring nitrofurantoin resistance in *Klebsiella* isolates, are shown in Table 3.

### 2.4. Description of Plasmids with Antibiotic-Resistance Genes

**Figure 4:**
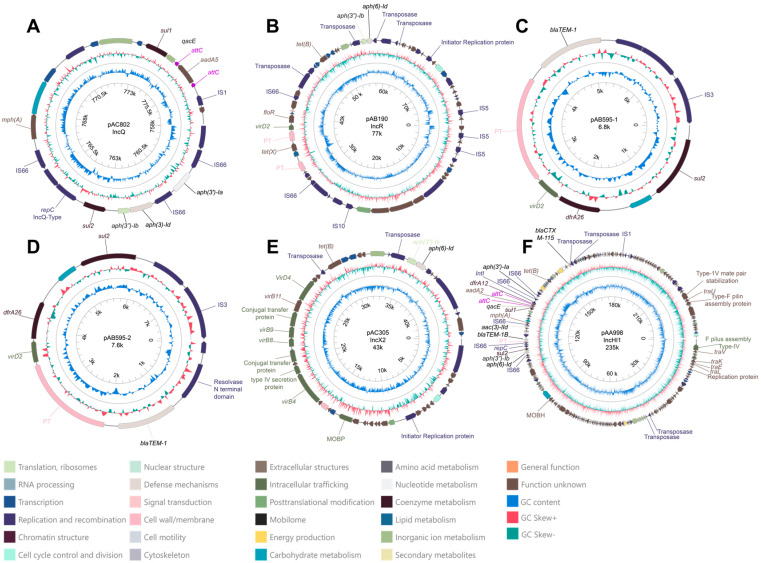
Graphical representation of plasmids containing ARGs, generated using the PlasmidScope web server. (**A**) Multi-drug-resistance region of plasmid pAC802 (*E. coli* A224-8), (**B**) plasmid pAB190 (*E. coli* A224-8), (**C**) plasmid pAB595-1 (*K. pneumoniae* A238-6), (**D**) plasmid pAB595-2 (*K. pneumoniae* A230-7), (**E**) plasmid pAC305 (*C. gillenii* A223-7), and (**F**) plasmid pAA998 (*E. coli* A231-12). Since plasmid pAC802 was found to be associated with a chromosomal contig after PacBio sequencing, we only present the multi-drug-resistance region integrated into the chromosome, which was linked to a plasmid sequence (from 758 to 777 kbp). In each graphical representation, all coding sequences (CDSs) identified by Prokka and Prodigal are displayed; however, only genes related to antibiotic resistance, biocide resistance, integrons, replication, insertion sequences (ISs), transposases, putative transposases (PTs), and other genes related with mobility functions are highlighted. Insertion sequence families were assigned based on MOB-suite results or annotated with the PlasmidScope web server. As *attC* sequences were not annotated with PlasmidScope, they were added manually based on genomic coordinates as indicated by IntegronFinder 2.0. Colors of CDSs in the plasmid maps are based on categorical functions assigned by eggNOG-mapper in PlasmidScope. Incompatibility groups (when available) and plasmid sizes were determined using the MOB-suite tool and are indicated in the graphics. To enhance visualization, all plasmid sequences are displayed in their circular form and gene names with light colors were changed to black. Additional details on these plasmids are provided in Supplementary Table S3.

In total, we detected 50 plasmids across the 15 sequenced genomes with the Illumina data (Supplementary Table S3). Site 3 presented the highest number of plasmids (23 in total); however, no statistically significant differences were observed between sites regarding plasmid numbers (Chi = 2.354; *p* = 0.308). Other statistical values associated with plasmid diversity are presented in Table 2. Interestingly, only 6 out of the 50 plasmids contained ARGs (Figure 4). All the plasmids were assigned to different incompatibility groups (when available) and exhibited variable sizes, except for plasmids pAB595-1 and pAB595-2, which were isolated from *K. pneumoniae* isolates at Sites 3 and 2, respectively. Both plasmids contained the same three ARGs (*blaTEM-1*, *dfrA26*, and *sul2*) but differed in size, with plasmid pAB595-2 being larger (7651 bp; Figure 4D) and containing additional coding sequences. Plasmids pAA998 and pAC802 were the only ones to possess an integron in their structure. However, while pAC802 contained an integron, it lacked an integrase gene and only carried three gene cassettes linked to antibiotic and biocide resistance (*sul1*, *aad5*, and *qacE*). In contrast, plasmid pAA998 presented a complete integron with three ARGs (*sul1*, *aadA2*, and *drfA12*) alongside a biocide resistance gene (*qacE*). Additionally, plasmids pAC802, pAB190, pAB595-1, pAB595-2, and pAA998 contained insertion sequences (ISs) within their structures, with some located near ARGs. These IS elements are highlighted in graphical representations due to their potential roles in ARG mobilization and expression [19,20]. Other transposases that could not be identified as belonging to any specific IS family are labeled as a “Transposase” or “Putative Transposase (PT)”.

#### 2.4.1. Plasmid pAC082

This putative plasmid was initially identified by MOB-suite in the assembly of *E. coli* A224-8 (Site 3) using only Illumina data. However, after reconstructing the genome through hybrid assembly (PacBio and Illumina), the sequence of this plasmid was found to be integrated into the chromosomal contig of *E. coli* A224-8 (Supplementary Table S3). The plasmid carried seven ARGs (*aph(3′)-Ia*, *aph(6)-Id*, *aph(3′)-Ib*, *sul2*, *mph(A)*, *sul1*, and *aadA5*), along with an incomplete class 1 integron (lacking the integrase gene) and four insertion sequences (ISs). A replication sequence associated with the IncQ1 incompatibility group was also identified (Figure 4A). The incomplete integron contains two gene cassettes associated with antibiotic resistance (*sul1* and *aadA5*) and one linked to biocide resistance (*qacE*), as annotated by the IntegronFinder 2.0 software [20] (Supplementary Table S4). This plasmid lacks genes associated with horizontal transfer mechanisms or relaxase activity, classifying it as a non-mobilizable plasmid. The closest match observed in the database by the MOB-suite v3.1.8 software was plasmid pASL01 from *E. coli* (mash distance: 0.051; coverage: 57%; identity: 99.88%) with a size of 27 kb.

#### 2.4.2. Plasmid pAB190

This plasmid was also observed in *E. coli* A224-8 and carried five ARGs (*tet(X)*, *tet(B)*, *aph(3″)-Ib*, *aph(6)-Id*, and *floR*), along with six ISs. Its replication sequence is associated with the IncR incompatibility group. Although genes coding for conjugative elements were not identified, a sequence corresponding to a MOBF-class relaxase was detected, indicating that the plasmid is likely mobilizable. The most similar plasmid in the databases was plasmid pEFER from *E. fergusonii* (mash distance: 0.017; coverage: 66%; identity: 99.46%) with an estimated size of 77 kb (Figure 4B).

#### 2.4.3. Plasmids pAB595-1 and pAB595-2

Two plasmids were associated with the primary AB595 cluster according to the MOB-suite tool. They were present in *Klebsiella pneumoniae* A238-6 (Site 3, plasmid pAB595-1) and *Klebsiella pneumoniae* A230-7 (Site 2, plasmid pAB595-2), with approximate sizes of 6.8 kb and 7.6 kb, respectively (Figure 4C,D). Although MOB-suite classified both plasmids as non-mobilizable due to the absence of replicase, relaxase, or conjugative elements, additional analysis using the PlasmidScope server identified a VirD2-related gene within their sequences. The presence of VirD2 (relaxase), a component commonly involved in conjugative transfer, suggests that these plasmids may have potential mobility despite their initial classification. Both plasmid sequences contained three ARGs (*bla_TEM-1_*, *sul2*, and *dfr26*), and an insertion sequence (IS) from the IS3 family. Both plasmids share the highest similarity with the *E. coli* plasmid RCS86_pI. For plasmid pAB595-1, we observed a mash distance of 0.036 with 47% coverage and 100% identity. Similarly, plasmid pAB595-2 exhibited a mash distance of 0.038 with 42% coverage and 100% identity.

#### 2.4.4. Plasmid pAC305

This plasmid was identified in the *C. gillenii* isolate A223-5 (Site 2) and has an estimated size of 43 kb. Its replication sequence is associated with the IncX2 group, and it harbors three ARGs (*tet(B)*, *aph(3)-Ib*, and *aph(3)-Id*). The plasmid contains all the necessary genes for the conjugation process, classifying it as a conjugative plasmid. The closest match in the database is the R6K plasmid from *E. coli* (mash distance: 0.015; coverage: 76%; identity: 99.99%).

#### 2.4.5. Description and Comparative Analysis of Plasmid pAA998

**Figure 5:**
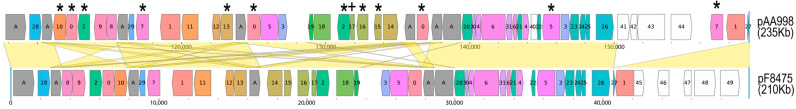
Comparative analysis of plasmid pAA998. Alignment of coding regions between plasmids pAA998 and pF8475, arranged based on their similarity in gene content (Jaccard index). Coding sequences (CDSs) that are unique to either plasmid are highlighted with a white background. CDSs associated with antibiotic-resistance genes are marked with an asterisk (*), while those related to quaternary-ammonium-compound resistance are indicated with a plus sign (+). For improved visualization, the section of plasmid pAA998 containing genes 32 to 39 (unique to pAA998) has been removed. Colors and numbers in the CDS boxes indicate associated functions, which are detailed further in Supplementary File S2.

The plasmid pAA998, found in the *E. coli* A231-12 isolate (Site 3), contained 12 ARGs, the highest number of all the plasmids. Of these ARGs, three (*sul1*, *aadA2*, and *drfA12*) were located within a class 1 integron, along with a biocide-resistance gene (*qacE*) (Supplementary Table S4). This integron included the integrase gene, whose product is responsible for mobilization and structural rearrangements. The integron is situated alongside nine other ARGs (*bla_CTXM-115_*, *mph(A)*, *aac(3)-IId*, *tet(B)*, *aph(6)-Id*, *aph(3″)-Ib*, *aph(3)-Ia*, and *sul2*) within the multidrug-resistance region, spanning 110 kb to 160 kb (Figure 4F and Figure 5). The plasmid is approximately 235 kb in size and carries two replicases from the IncH and IncQ1 groups (Supplementary Table S3). It also contains six insertion sequences (ISs), as well as all the necessary genes for conjugation, classifying it as a conjugative plasmid. The closest match in the database was plasmid pF8475 from *Salmonella enterica* (mash distance: 0.003, coverage: 89%, identity: 99.98%).

Given the significance of IncH plasmids in ARG exchange within the Enterobacteriaceae family [21], we conducted further analyses to examine the genomic structure and relationships of pAA998. Specifically, we compared its complete structure with that of its closest relative matched in the MOB-suite database, plasmid pF8475, which is derived from an environmental isolate of *Salmonella enterica*. The alignment of the two plasmids is shown in Figure 5 While most coding sequences (CDSs) are conserved between the two plasmids, notable rearrangements such as inversions and translocations occur, even within the multidrug-resistance region. Full annotations of the CDSs based on COG and Pfam are provided in Supplementary File S2.

## 3. Discussion

In this work, we determined that as water quality in the river declined, the richness and complexity of ARGs in Enterobacterial plasmids increased. Human activities are strongly impacting plasmid dynamics and, therefore, also the dissemination of ARGs in natural environments. In this context, our results underscore the urgent need to expand antimicrobial-resistance surveillance to natural environments with both low and high human impact due to their significant implications for environmental and public health.

Seven isolates presented a resistance to at least 1 of the 15 antibiotics tested, with two classified as multidrug-resistant (resistant to three or more antibiotics) [15]. Of those seven, five showed resistance to nitrofurantoin, including those classified as *Klebsiella pneumoniae* (A231-5, A237-6, A230-7, and A238-6) and as *K*. *quasipneumoniae* (A230-5). *Klebsiella* isolates have been documented to frequently exhibit high resistance levels to nitrofurantoin, with resistance observed in 69.2% of evaluated isolates. This indicates a high prevalence of nitrofurantoin resistance in bacteria from this genus, which appears to be sustained in our isolates from a polluted river.

This antibiotic was introduced in 1953 as a first-line treatment for urinary tract infections and was widely used for two decades. Nonetheless, it was replaced mainly by trimethoprim/sulfamethoxazole and β-lactam antibiotics as these alternatives became more commonly employed [22]. However, the increasing resistance to trimethoprim/sulfamethoxazole and other antibiotics in clinical bacteria has led to a resurgence in its use in recent decades, which may be contributing to a rise in resistance to this antibiotic in environmental isolates [23,24].

The development of resistance to nitrofurantoin is primarily linked to mutations in the *nfsA* and *nfsB* genes and deletions in the *ribE* gene [25]. Likewise, resistance to this antibiotic has also been associated with the presence of efflux pumps of the RND family that are encoded by *oqx* genes mobilized on plasmids or inserted into the chromosome [22,26]. We observed multiple variants of the *oqx* genes in the chromosome of these isolates and different mutations in the *nfsA* gene (Table 3). This suggests that the E29A, G125W, G204S, Q195L, and K222R mutations associated with *oqx* genes could be involved in developing nitrofurantoin resistance in *Klebsiella* isolates. Notably, the high proportion of *Klebsiella* isolates exhibiting resistance to this antibiotic is noteworthy. Future studies should explore whether the environmental incidence of nitrofurantoin-resistant bacteria is associated with the high rates of prescription [27] and subsequent release of this antibiotic into the environment.

We also identified three isolates resistant to trimethoprim/sulfamethoxazole, a combination of antibiotics commonly used to treat urinary tract infections and various other infections [28]. One of these isolates (*K. pneumoniae* A230-7) was from Site 2, while the other two (*E. coli* A231-12 and *K. pneumoniae* A238-6) were obtained from Site 3. Furthermore, both trimethoprim and sulfamethoxazole were detected in water samples from Sites 2 and 3, with no presence in Site 1 (see Supplementary Table S5 for detected concentrations).

All the isolates resistant to trimethoprim and sulfamethoxazole contained the *dfrA* and *sul* genes on plasmids, which confer resistance to these respective antibiotics. Additionally, we observed the *sul1* and *dfrA12* genes in a class 1 integron (plasmid pAA998) in isolate *E. coli* A231-12. This finding aligns with other studies that consistently report a correlation between class 1 integrons and trimethoprim/sulfamethoxazole-resistance genes [29,30].

The presence of *sul* and *dfrA* genes, particularly within mobile genetic elements like plasmids and integrons in environmental isolates, may be linked to the presence of antibiotic residues in the aquatic environment, given that relevant concentrations of sulfamethoxazole and trimethoprim were detected only in Sites 2 and 3. Similar patterns have been observed in other studies, where aquatic sources that presented higher concentrations of sulfonamide residues (due to wastewater discharges) also exhibited a greater abundance of sulfonamide-resistant bacteria and *sul* genes [31,32].

In the same integron of plasmid pAA998, we detected the genes *qacE* and *aadA2*, which confer resistance to quaternary ammonium compounds (QACs) and streptomycin, respectively. This is notable, as the *qacE* gene was also found within the integron structure of *E. coli* isolate A224-8, another isolate from Site 3. Wastewater treatment plants commonly use QACs to reduce bacterial loads in wastewater [33]. Site 3, positioned downstream of a municipal wastewater treatment plant (see Supplementary Figure S1 and Section 4), likely receives these compounds, potentially explaining the presence of the *qacE* gene in these isolates. Detecting genes related to both QAC and antibiotic resistance within the same plasmids and integrons is highly relevant, as this co-localization may facilitate co-resistance in bacteria [34]. Therefore, future environmental monitoring of ARGs should include markers for these genes to better assess their co-existence and potential co-selection events.

Notably, *E. coli* A231-12, isolated from the most polluted site, showed resistance to seven antibiotics, including ampicillin/sulbactam and all the cephalosporins tested (Figure 2). It harbored two ARGs associated with beta-lactam antibiotic resistance; the *bla_CTX-M-115_* gene, which belongs to the class A extended-spectrum beta-lactamases, and the *bla_TEM-1_* gene, which encodes the class A broad-spectrum beta-lactamase TEM-1. According to the classification of Bush and Jacoby [35], the enzymes encoded by the gene families *CTX* and *TEM-1* are classified in the groups 2b and 2be, respectively. Group 2b confers a phenotype resistant to penicillin and some of the first-generation cephalosporins, while group 2be is resistant to an extended spectrum of cephalosporins. Both genes, *bla_CTX-M-115_* and *bla_TEM-1_*, were found in the conjugative plasmid pAA998, and the *bla_TEM-1_* gene was also observed in the plasmids pAB595-1 and pAB595-2 (*K. pneumoniae* isolates A238-6 and A230-7, respectively) suggesting that these beta-lactamases might be spread to other bacteria in the environment.

In a developing country like Costa Rica, extended-spectrum beta-lactamase (ESBL)-producing bacteria have been documented in both clinical and environmental settings [36,37], with TEM-type ESBL producers consistently observed and CTX-type ESBL producers found less frequently [36]. Continuous monitoring of these bacteria is a core part of the country’s National action plan to combat antimicrobial resistance, highlighting the importance of tracking ESBL-producing bacteria in natural and clinical settings [36].

The presence of genes encoding ESBLs in aquatic environments due to anthropogenic pollution, particularly sewage, has been widely studied in different regions; however, available published information is scarce for Latin America [37,38,39]. These genes pose a significant threat in hospital settings, where bacteria may carry them chromosomally or acquire them through plasmids, leading to multidrug resistance and complicating infection treatment. Detecting bacteria with these plasmids in community-accessible aquatic environments highlights the need for active ARG monitoring in natural settings, as they can serve as reservoirs of resistance genes relevant to treating infections in humans and animals. Thus, generating data on the prevalence of ESBL-positive isolates and identifying plasmid-borne *bla_TEM-1_* and *bla_CTX_* genes in natural environments is crucial for ongoing monitoring efforts and risk assessment in Costa Rica and worldwide.

The plasmid pAA998, which harbored the higher number of ARGs, exhibited a conserved structure compared to its closest match in the MOB-suite database, plasmid pF8475; nevertheless, different genes are only present in pAA998 or in pF8475 (full annotations are listed in Supplementary File S2). Similarly, two ARGs present in plasmid pAA998 (*aph(3′)-Ia* and *bla_CTX-M-115_*) are absent in pF8475, indicating that despite the strong genomic similarity between the two plasmids, their structures have undergone modifications. These changes are particularly evident in the multidrug-resistance region where various translocations and reversions are evident (Figure 5).

IncH plasmids are among the largest plasmids carrying antibiotic-resistance genes within the Enterobacteriaceae family [21], representing a potential risk as mobilizable elements that can acquire ARGs in the environment and transfer them to clinical settings. Both plasmids, pF8475 and pAA998, were found in environmental isolates of Enterobacteriaceae (*Salmonella enterica* and *Escherichia coli*, respectively) in two distanced countries (Czech Republic and Costa Rica). Although they share a high percentage of sequence identity, structural rearrangements and differences in their gene composition suggest that they are distinct plasmids with a common ancestral origin. Furthermore, the substantial presence of ARGs within these plasmids likely supports the fact that although the presence of ARGs in plasmids can be linked to their evolution under antibiotic pressure [40], the persistence of plasmids carrying ARGs in free-living bacteria is also highly frequent [40,41].

The mobility potential of plasmid pAA998 to other bacteria is notable, as the MOB-suite tool predicted it to be conjugative due to the presence of genes associated with the type IV secretion system (Figure 4F). Similarly, plasmid pAC305 exhibited conjugative elements in its structure, along with three antibiotic-resistance genes (Figure 4E). Otherwise, plasmid pAB190, which harbors five ARGs (Figure 4B), was classified as a mobilizable plasmid because it lacks conjugative elements but presented a relaxase gene. However, it was found in the *E. coli* isolate A224-8, which also contains other conjugative plasmids (Supplementary Table S3) that could facilitate the mobilization of pAB190 and its associated ARGs [42].

While plasmids pAB595-1, pAB595-2, and pAC802 were classified as non-mobilizable due to the absence of relaxase and conjugative elements in their sequences, this classification may be influenced by the MOB-suite limitations [43]. Notably, the presence of a VirD2-related gene in pAB595-1 and pAB595-2, as identified by PlasmidScope (which utilizes eggNOG-mapper), suggests a potential for mobility that might have been overlooked by MOB-suite. Reconstructing plasmid sequences from draft genomes is inherently challenging, and certain mobility-associated features may remain undetected. Nonetheless, our findings highlight the potential for highly contaminated environments to harbor plasmids with resistance genes that may be transferred to other relevant bacteria, such as species within the Enterobacteriaceae family. However, further studies are needed to detect specific plasmid sequences using more sensitive techniques, such as qPCR, digital PCR, or metagenomic approaches, to understand these dynamics better.

Although no plasmid-borne ARGs were observed in Site 1, the presence of the *blaEC* gene in all five *E. coli* genomes isolated from this site, an area with relatively low human impact, represents a notable finding. The *blaEC* genes, also known as *ampC-EC* genes, are primarily chromosomally encoded and associated with *Escherichia coli* and *Shigella* strains [44,45]. Specifically, the *blaEC-15* gene is linked to *E. coli* phylogroup A, while the *blaEC-18* gene is associated with phylogroup B1 [45]. This aligns with our results, where isolate A229-5 from Site 1, which contains the *blaEC-15* gene, belongs to phylogroup A, whereas isolates A236-7, A229-12, and A222-7 from Site 1, as well as A231-12 from Site 3, harbor the *blaEC-18* gene and are classified into phylogroup B1.

However, none of the *E. coli* isolates from Site 1 exhibited phenotypic resistance to the antibiotics tested, even though the *blaEC-15* and *blaEC-18* genes have been reported as beta-lactamases with extended activity against cephalosporins [44,45]. It has been suggested that, despite the numerous variants of these genes, only some of them are expressed and confer a resistant phenotype to beta-lactam antibiotics. The effectiveness of these genes has been linked to the presence of nearby insertion sequence promoters [44] as well as their interactions with efflux pumps and low outer-membrane permeability [46]. Furthermore, the horizontal gene transfer of *ampC* genes has gained significance in recent years, as it has been associated with the clonal expansion of resistance strains [47], highlighting the need for continuous monitoring of these genes [48].

The detection of ARGs in environments with low human impact, such as Site 1, represents a novel and necessary strategy. These environments may serve as reservoirs of resistance genes that could later contribute to the broader dissemination of resistance traits. This also emphasizes the role of chromosomal ARGs, which can persist and potentially evolve within isolated bacterial populations, conferring resistance phenotypes. Thus, future studies in areas distant from human influence may further illuminate the origins and dynamics of chromosomally and plasmid-encoded ARGs, enhancing our understanding of how isolated reservoirs contribute to the global resistome.

Site 3 was chosen as a reference point in the Virilla River’s contamination gradient, as it has been previously reported to have the highest pollution levels according to the BMWP-CR index (values between 17; classified as “very polluted”) and the Dutch Quality Index (value of 11; classified as “severe”) [49]. Additionally, it exhibited the lowest NSFWQI value obtained in this work (23; very poor quality; Supplementary Table S5). However, given the intensive effort required to assess microbiological pollution levels, all three sites may pose a potential risk to human health. Future studies should incorporate additional intermediate sites and expand the evaluation to cover a broader range of microbiological parameters and biological data, providing a more comprehensive understanding of this potential risk.

As mentioned above, the Virilla River is impacted by wastewater discharged from a treatment plant located between Site 2 and Site 3 (see Supplementary Figure S1 and Section 4). This discharge is likely a primary contributor to the elevated contamination levels observed at Site 3 and may be a significant factor in the presence of ARGs and multidrug-resistant bacteria, as wastewater treatment plants are known hotspots for the dissemination of antimicrobial-resistance traits [50]. Although our sample size is limited, discovering the highest number of ARGs and plasmids in isolates from Site 3 raises concerns about the potential selective pressure exerted by urban pollution (including sewage), which may enhance the transfer and distribution of plasmids carrying ARGs, as previously discussed. Similar patterns have been observed globally, such as in coastal regions of central Thailand, where urban pollution appears to drive the high prevalence of ARGs and mobile genetic elements in the aquatic environment [51]; likewise, in aquatic environments of the Bolivian Andes, a significant correlation was found between fecal contamination and an increase in ARGs as well as the presence of the *intl1* gene [52].

This relationship between urban contamination and the prevalence of ARGs is supported by experiments on microbial communities (microcosms) such as the one carried out by Aoife and Rima [53], where antibiotic-resistant bacteria proliferated more effectively in microcosms treated with wastewater than those treated only with river water; demonstrating that bacteria with ARGs that survive wastewater treatment can proliferate and persist in the aquatic environment after their release. Additionally, a study evidenced that in microcosms created from wastewater from a treatment plant in Scandinavia, conjugation rates were increased upon exposure to low concentrations of antibiotics and biocides [54]. These findings emphasize the importance of conducting further studies in various lotic water bodies, particularly those under strong urban influence.

Another noteworthy point is the isolation of multiple *E. coli* strains from all three sampling sites, each displaying distinct ARG profiles. In Figure 1, we highlight how isolates *E. coli* A224-8 and *E. coli* A231-12, both from Site 3, exhibit a significantly higher number of ARGs, due to the presence of plasmids, compared with other *E. coli* isolates from Sites 1 and 2, which belong to the same clades. This observation suggests that, despite sharing evolutionary origins within the same phylogroups, the acquisition of plasmid-borne ARGs occurs only in the isolates from Site 3, supporting the premise that the highly contaminated environment at Site 3 may facilitate the acquisition of new plasmids carrying resistance genes.

## 4. Materials and Methods

### 4.1. Sampling Sites

Field trips were conducted during the rainy season of 2021 to three locations along the Virilla River Watershed (Supplementary Figure S1). Site 1, near the river’s source in Vásquez de Coronado (9°59′9″ N, 83°56′35″ W), shows minimal human impact and is situated at the highest elevation at an altitude of 2020 m. Site 2, located in Tibás (9°57′47″ N, 84°5′51″ W), is at an altitude of 1185 m and has a high population density with significant environmental transformation. Site 3 is located at the Brasil Dam in Mora (9°56′46″ N, 84°13′22″ W), where the Virilla River converges with the Uruca River. Positioned at an altitude of 720 m, this site is downstream of the ”Los Tajos” treatment plant, which discharges wastewater directly into the Virilla River. Although it experiences moderate human impact, it recorded the lowest water quality among the sites. For the three sites, the National Sanitation Foundation Water Quality Index (NSFWQI) value was calculated [48], confirming that the surface water quality degraded during the rainy season of 2021 (poor quality for Sites 1 and 2 and very poor quality for Site 3) (Supplementary Table S5). Microbiological parameters of surface water samples collected during the rainy season (November 2021) were measured with the most-probable number technique, together with measures of conductivity, turbidity, color, and pH; other physical–chemical variables were analyzed in the field with a multiparameter probe (YSI, model 85 A, Yellow Springs, OH, USA) (Supplementary Table S5). Indices from the Costa Rica Ministry of Environment support these data and can be verified in the National Information System for Integrated Water Resource Management (SINIGIRH) [49].

### 4.2. Sample Processing and Bacterial Isolation

Surface water samples were collected on three separate days from the designated sites using sterile containers and processed on the same day. Initially, 250 mL of each sample was prefiltered through a sterile 80 µm pore glass fiber prefilter (Sartorius, Göttingen, Germany). Subsequently, each prefiltered sample was passed through a sterile 0.2 µm pore filter (Sartorius). The filters were then incubated at room temperature in 40 mL of Lauryl broth (Difco-BBL, Franklin Lakes, NJ, USA) for 24 h in aerobic conditions to promote the growth of fecal coliforms. After incubation, the Lauryl broth (BBL) was transferred to culture plates containing Levine (Oxoid, ThermoFisher™, Waltham, MA, USA) and MacConkey agar (Oxoid), where the colonies were incubated at 35 °C for 24 h. These media facilitate the differentiation of various morphotypes within the Enterobacteriaceae family. Colony-forming units from different sampling points were identified, and morphotypes were transferred to new culture media to obtain pure cultures. These morphotypes were stored in duplicate 1.5 mL tubes containing 20% glycerol in Soy Tryptose Broth (Oxoid) after 24 h of growth and preserved at −80 °C.

### 4.3. Biochemical and Serological Analysis

Cryopreserved bacteria were inoculated onto MacConkey agar (Oxoid) and incubated for 24 h at 35 °C. After incubation, at least one lactose-positive morphotype from each sample was isolated and subcultured onto tryptic soy agar plates (Oxoid). Following another 24 h incubation at 35 °C, the API20E test (Biomerieux, Marcy-l’Étoile, France) was performed according to the manufacturer’s instructions. Each bacterial isolate was identified based on its numerical profile using the apiweb™ v1.3.1 software. The Wellcolex *Shigella* color kit (ThermoFisher™, Waltham, MA, USA) was used for the detection and species identification of Enterobacteria genomically related to *Shigella* spp., following the manufacturer’s instructions.

### 4.4. Chemical Analysis

Water samples were collected in 1 L amber glass containers and transported under appropriate conditions to the laboratory at the Center for Research in Marine Sciences and Limnology (CIMAR) at the University of Costa Rica for nutrient analysis (nitrite, ammonium, nitrate, phosphate, and silicate; Supplementary Table S5) [55,56].

### 4.5. Anthropogenic Contaminant Detection by Mass Spectrometry

Detection analyses of caffeine, terbutryn, and various antibiotics, including amoxicillin, ciprofloxacin, clarithromycin, doxycycline, enrofloxacin, erythromycin, lincomycin, oxytetracycline, penicillin V, sulfadiazine, sulfamethazine, sulfamethoxazole, sulfathiazole, tetracycline, tiamulin, trimethoprim, tylosin, chlortetracycline, levofloxacin, chlorhexidine, and moxifloxacin, in water samples were conducted at the Central American Institute for Studies on Toxic Substances (IRET) Laboratory at the National University of Costa Rica (UNA). The analyses utilized direct injection and LC-MS/MS measurements, employing an ACQUITY H-CLASS UPLC chromatographic system (Waters Corp., Milford, MA, USA) connected to a Xevo TQ-S triple quadrupole mass spectrometer (Waters Corp., Wilmslow, Manchester, UK), which was equipped with an electrospray ionization interface operating in positive mode. Only detected contaminants are reported in Supplementary Table S5.

### 4.6. DNA Extraction and Sequencing

From the total collection of isolated bacteria (72 isolates), 15 were randomly selected for sequencing (5 from each sampling site). Genomic DNA was extracted using the DNeasy PowerSoil kit (Qiagen, Venlo, The Netherlands) following the manufacturer’s instructions, and samples were sequenced at Novogene using the Illumina NovaSeq platform (Hong Kong, China), yielding at least 1 GB of paired-end raw data per genome.

### 4.7. Quality Control of Raw Data

Raw data were processed with the BBduk function from the BBtools package [57] to remove remaining adapter sequences, and Trimmomatic [58] was employed to trim the first ten nucleotides due to uneven tetranucleotide frequencies. Sequences with average quality scores below 30 or lengths less than 100 bp were discarded.

### 4.8. Assembly and Quality Evaluation

Assembly was conducted using Spades v3.15.4 [59], with k-mer values of 21, 33, 55, 77, and 99. Contigs shorter than 1000 bp were removed, and assembly statistics were visualized with Quast [60]. The completeness and contamination of genomes were assessed with the checkm2 v1.0.2 software [61].

### 4.9. Taxonomic Identity and Phylogenomic Analysis

Taxonomic identity was determined using the Type (Strain) Genome Server (TYGS) [13]. For isolates not recognized by the TYGS tool, we employed the Genome Taxonomy Database (GTDB) and the neighbor-joining method to identify closely related genomes [62]. Additionally, we conducted a genome-wide phylogenomic analysis that included our genomes alongside 79 additional genomes downloaded from the GenBank database. Genomes were selected based on criteria such as their status as type strains, their representation of various phylogroups or subclades identified in previous studies [63,64,65,66], and reference genomes from NCBI. The selected genomes were imported into Anvi’o for coding sequence identification using Prodigal [67], followed by amino acid alignment with DIAMOND [68] and gene cluster creation using the MCL algorithm [69]. We then selected and concatenated the nucleotide sequences of all single-copy core genes (SCCGs) across all the genomes, which resulted in a dataset of 908 SCCGs. The nucleotide sequences of these genes were aligned using MAFFT v7.397 [70], and a maximum likelihood analysis was performed with FastTree v2.1.10 (GTR model with 1000 bootstrap replicates) [71]. The resulting phylogenomic tree was visualized and edited using iTOL [72].

### 4.10. Antibiotic Susceptibility Testing

Antibiotic-resistance profiles were determined by culturing isolates on LB agar for 24 h, followed by analysis with the VITEK2 system. The antibiotic susceptibility card VITEK^®^ 2 AST-N401 (Biomerieux, Marcy-l’Étoile, France) included aminoglycosides (amikacin, gentamicin), β-lactams (ampicillin/sulbactam), carbapenems (ertapenem, meropenem), cephalosporins (cephalothin, cefazolin, ceftazidime, ceftriaxone, cefepime), quinolones (ciprofloxacin, norfloxacin), sulfonamides/diaminopyrimidines (trimethoprim/sulfamethoxazole), and others such as fosfomycin and nitrofurantoin. Resistance levels were assessed based on the MIC breakpoints for Enterobacterales as outlined in the 2020 CLSI guidelines [16].

### 4.11. Identification of Antibiotic-Resistance Genes (ARGs) and Mobile Genetic Elements (MGEs)

Plasmids were reconstructed using MOB-suite v3.1.8 [73], which facilitated the separation of contigs associated with each plasmid from chromosomal sequences. Integrons were mapped utilizing Integron Finder 2.0 [74] to ascertain their genomic or plasmid contexts. Potentially reconstructed plasmids were annotated and visualized with the PlasmidScope v1.2 web server [75], incorporating coding sequences identified with Prokka v1.14.5 [76], and Prodigal v2.6.3 [67] and annotated with eggNOG-mapper v2 [77] but only highlighting the antimicrobial-resistance genes (ARGs), integrons, replicon sequences, insertion sequences (ISs) and other genes related to mobility.

Antimicrobial-resistance genes were identified using the ABRicate v0.9.8 tool [78], which employed the NCBI AMRfinderPlus database (updated to 1 November 2024) [79], applying a minimum coverage and similarity threshold of 80%. A Kruskal–Wallis test was conducted with a confidence level of 95% in R software (version 4.4.3) to evaluate significant differences in the abundance of ARGs and plasmids across sampling sites. Subsequently, a post hoc Dunn test was performed to determine specific sites exhibiting significant differences for each variable.

### 4.12. Search for Mutations Conferring an Antibiotic-Resistance Phenotype

All genomes were annotated using Prokka v1.14.6 [22], followed by manual searches for point mutations in specific resistance-related genes. The gyrA gene from susceptible *E. coli* K-12 substr. MG1655 (reference genome GCF_000005845.2) was used as a reference to compare amino acid sequences against the genome of isolates classified as *E. coli*. The same analysis was conducted for *nfsA* and *nfsB* genes associated with nitrofurantoin resistance in genomes of isolates classified as *Klebsiella*, comparing them against the reference strain *Klebsiella pneumoniae* ATCC 13883 (accession number JOOW01000000).

### 4.13. Sequencing of A224-8 and A231-12 Isolates Through PacBio Technology for Plasmid Confirmation

To confirm the presence of plasmids with ARGs, the genomes of two isolates were sequenced using PacBio technology. This approach aimed to improve the assembly quality and validate the plasmid structures, as plasmids often contain repetitive sequences exceeding 300 bp, which are challenging to assemble with Illumina data [80]. Samples were sent to Novogene for long-read sequencing, and hybrid assembly was performed with Spades v13.5.4. Subsequent analyses to annotate ARGs and plasmids employed the previously mentioned tools.

### 4.14. Genomic Comparison of Plasmid pAA998

The plasmid pAA998 belongs to the incompatibility group IncH and was identified in isolate A231-12, which harbors 12 antibiotic-resistance genes (ARGs). Given the importance of this incompatibility group in disseminating antibiotic resistance and the substantial number of ARGs present in its structure [21], we conducted a sequence comparison with the most closely related plasmid in the MOB-suite database, pF8475 (accession number KP899804). We annotated both plasmids using Anvi’o v7.1, employing the COG14 [81] and Pfam v32 [82] databases to achieve this. Alignments were generated and visualized following the PlasX-MobMess pipeline [83].

## 5. Conclusions

This study highlights the critical connection between urban pollution and the rise of antibiotic resistance in aquatic environments. Our findings reveal that both genotypic and phenotypic resistance levels were highest at more urbanized sites, with common resistance to nitrofurantoin, trimethoprim/sulfamethoxazole, and beta-lactams linked to the *sul*, *dfrA*, and *bla* genes present in mobile genetic elements, such as plasmids and class 1 integrons. Notably, an *E. coli isolate* from the most polluted site showed the highest ARG count on a single plasmid, classified within the IncH incompatibility group, which signals a strong potential for ARG transfer within Enterobacteriaceae. The observed increase in resistance complexity and abundance along the urban gradient underscores the need for antimicrobial-resistance surveillance within water quality monitoring to help mitigate the transfer of resistance traits between environmental and community settings.

## Figures and Tables

**Table 1:** Characteristics of the assembled genomes. Size, N50, L50, and GC content were determined with Quast; the closest taxonomy was obtained using the Type Strain Genome Server and the Completeness and Contamination with Checkm2.

Isolate (Site)	Sequencing Method	Contigs	Size (Mb)	N50	L50	%GC	Completeness	Contamination	Taxonomy According to TYGS (dDDH in %)
A222-7 (1)	Illumina	73	4.90	130,684	11	50.61	100	0.05	*Escherichia coli* (73.8)
A229-5 (1)	Illumina	88	4.86	111,924	14	50.76	100	0.04	*Escherichia coli* (73.8)
A229-12 (1)	Illumina	99	4.75	103,479	15	50.78	100	0.09	*Escherichia coli* (74.7)
A236-7 (1)	Illumina	64	4.80	167,951	10	50.7	100	0.15	*Escherichia coli* (74)
A236-12 (1)	Illumina	73	4.7	147,946	8	50.59	100	0.19	*Escherichia coli* (75.1)
A223-7 (2)	Illumina	45	4.97	413,561	6	52.48	100	0.05	*Citrobacter* sp. (42.5) *
A230-5 (2)	Illumina	40	5.41	296,086	6	57.74	100	0.09	*Klebsiella quasipneumoniae* (71.4)
A230-7 (2)	Illumina	50	5.48	349,092	6	57.22	100	0.23	*Klebsiella pneumoniae* (93.4)
A230-8 (2)	Illumina	159	4.96	77,565	23	50.62	100	0.12	*Escherichia coli* (74.1)
A237-6 (2)	Illumina	67	5.22	188,161	10	57.56	100	0.85	*Klebsiella pneumoniae* (93.4)
A224-7 (3)	Illumina	84	5.60	141,929	14	57.21	100	0.13	*Klebsiella variicola* (92.2)
A231-5 (3)	Illumina	51	5.47	215,170	9	57.27	100	0.26	*Klebsiella pneumoniae* (93.5)
A238-6 (3)	Illumina	75	5.54	191,124	10	57.19	100	0.19	*Klebsiella pneumoniae* (93)
A224-8 (3)	Illumina and PacBio	5	4.70	4,712,205	1	50.73	100	0.04	*Escherichia coli* (75.2)
A231-12 (3)	Illumina and PacBio	4	5.08	4,757,699	1	50.61	100	0.02	*Escherichia coli* (73.8)

* dDDH was calculated against the *Citrobacter tructae* genome (closest match).

**Table 2:** Minimum, median, maximum, total abundance, and richness (unique ARGs or plasmids) values of ARGs and plasmids observed in genomes from the three sampling sites. The Kruskal–Wallis test revealed statistically significant differences in the number of ARGs between sites (χ^2^ = 9.95; *p* = 0.007) but not in the number of plasmids (χ^2^ = 3.588; *p* = 0.166). Only results from Illumina data were used in the comparisons.

Site	Statistic	ARGs	Plasmids
1	Minimum	1	1
	Median	1	2
	Maximum	1	4
	Abundance	4	11
	Richness	3	11
2	Minimum	1	1
	Median	4	2
	Maximum	7	6
	Abundance	20	16
	Richness	19	16
3	Minimum	4	2
	Median	7	4
	Maximum	13	7
	Abundance	41	23
	Richness	29	23

**Table 3:** Mutations and *oqx* genes associated with nitrofurantoin resistance among isolates identified as *Klebsiella*. The genome of the susceptible reference strain *Klebsiella pneumoniae* ATCC 13883 (accession number JOOW01000000) was used for SNP detection.

Isolate (Site)	Phenotype	*nfsA* Mutations	*nfsB* Mutations	*oqx Genes*
*K. pneumoniae* A238-6 (3)	Resistant	E29A, G125W, G204S		*oqxB*, *oqxA*
*K. pneumoniae* A231-5 (3)	Resistant	Q195L		*oqxB11*, *oqxA5*
*K. pneumoniae* A237-6 (2)	Resistant	K222R		*oqxB25*, *oqxA6*
*K. pneumoniae* A230-7 (2)	Resistant	E29A, G125W, G204S		*oqxA*, *oqxB*
*K. quasipneumoniae* A230-5 (2)	Resistant	E29A, Q94E, Q147K, E191G, E194D	Q181K	*oqxA10*, *oqxB11*

## Data Availability

All generated data are available in the NCBI database under the Bioproject accession number PRJNA1164306. Illumina sequence reads can be retrieved with the accession numbers SRR30782601: SRR30782617. Other accession codes for additional data used are available in the supplementary tables. All codes employed in this study are available from the corresponding author upon request.

## Supplementary Materials

The following supporting information can be downloaded at: https://www.mdpi.com/article/10.3390/antibiotics13111089/s1, Supplementary Figure S1: Geographic map showing the location of the sampling sites (highlighted in red dots) and the wastewater treatment plant “Los Tajos” (yellow dot); Supplementary Figure S2: Tree inferred with FastME 2.1.6.1 from GBDP distances calculated for genome sequences obtained from the Type Strain Genome Server. The branch lengths are scaled in terms of the GBDP distance formula d5. The above branches are GBDP pseudo-bootstrap support values > 60% from 100 replications, with an average branch support of 34.8%. The tree was rooted at the midpoint; Supplementary Table S1: Accession numbers of the genomes used to reconstruct the phylogenomic tree illustrated in Figure 1; Supplementary Table S2: Complete annotation of antibiotic-resistance genes by the ABRicate tool employing the NCBI database; Supplementary Table S3: Plasmid annotation results obtained with MOB-suite software v3.1.8; Supplementary Table S4: Integron Finder annotation results for isolates A224-8 and A231-12; Supplementary Table S5: Physical–chemical and microbiological parameters used to calculate the NSFWQI of surface water samples during the rainy season (November 2021) in the Virilla River Watershed. Additionally, it includes anthropogenic contaminants measured by mass spectrometry. Supplementary File S1: Excel file containing all the supplementary tables; Supplementary File S2: Complete annotation of coding sequences found in plasmids pAA998 and pF8475 using the COG and Pfam databases.
