# Supplementary material for: The Impact of Urban Pollution on Plasmid-Mediated Resistance Acquisition in Enterobacteria from a Tropical River
Source: Antibiotics (Basel). 2024 Nov 14;13(11):1089. doi: 10.3390/antibiotics13111089 (PMC11591392; doi:10.3390/antibiotics13111089)
Supplement: Supplementary file 1 [file antibiotics-13-01089-s001.zip › Supplementary File S2.pdf]

accession	accession_representative	contigs	COG14_FUNCTION	Pfam
A	PF00665.26	2	COG2801:Transposase_InsO_and_inactivated_derivatives COG3316:Transposase_(or_an_inactivated_derivative)	PF00665.26:Integrase_core_domain PF13384.6:Homeodomain-like_domain PF13518.6:Helix-turn-helix_domain PF13551.6:Winged_helix-turn_helix PF13610.6:DDE_domain PF03050.14:Transposase_IS66_family_ PF09039.11:Mu_DNA_binding,_I_gamma_subdomain
0	PF01636.23	2	COG3231:Aminoglycoside_phosphotransferase COG3173:Predicted__kinase,_aminoglycoside_phosphotransferase_(APT)_family	PF01636.23:Phosphotransferase_enzyme_family
1	PF01609.21	2	COG3039:Transposase_and_inactivated_derivatives,_IS5_family COG3385:IS4_transposase	PF01609.21:Transposase_DDE_domain PF13751.6 PF05598.11:Transposase_domain_(DUF772) PF13701.6:Transposase_DDE_domain_group_1
2	COG0294	2	COG0294:Dihydropteroate_synthase	PF00809.22:Pterin_binding_enzyme
3	COG1309	2	COG1309:DNA-binding_transcriptional_regulator,_AcrR_family	PF00440.23:Bacterial_regulatory_proteins,_tetR_family PF02909.17:Tetracyclin_repressor-like,_C-terminal_domain
4	COG0789	2	COG0789:DNA-binding_transcriptional_regulator,_MerR_family	PF13411.6:MerR_HTH_family_regulatory_protein PF00376.23:MerR_family_regulatory_protein PF09278.11:MerR,_DNA_binding
5	PF07690.16	2	COG2211:Na+/melibiose_symporter_or_related_transporter COG2814:Predicted_arabinose_efflux_permease,_MFS_family	PF07690.16:Major_Facilitator_Superfamily PF12832.7:MFS_1_like_family
6	PF00403.26	2	COG1249!!!COG2608:Pyruvate/2-oxoglutarate_dehydrogenase_complex,_dihydrolipoamide_dehydrogenase_(E3)_component_or_related_enzyme!!!Copper_chaperone_CopZ COG2608:Copper_chaperone_CopZ	PF00403.26:Heavy-metal-associated_domain PF01134.22:Glucose_inhibited_division_protein_A PF03486.14:HI0933-like_protein PF13241.6:Putative_NAD(P)-binding PF00070.27:Pyridine_nucleotide-disulphide_oxidoreductase PF07992.14 PF13738.6 PF02852.22:Pyridine_nucleotide-disulphide_oxidoreductase,_dimerisation_domain
7	COG2367	2	COG2367:Beta-lactamase_class_A	PF00144.24:Beta-lactamase PF13354.6:Beta-lactamase_enzyme_family PF00905.22:Penicillin_binding_protein_transpeptidase_domain
8	COG3598	2	COG3598:RecA-family_ATPase	PF13481.6:AAA_domain
9	COG5527	2	COG5527:Protein_involved_in_initiation_of_plasmid_replication	PF06504.11:Replication_protein_C_(RepC)
10	COG3570	2	COG3570:Streptomycin_6-kinase	PF04655.14:Aminoglycoside/hydroxyurea_antibiotic_resistance_kinase
11	COG3440	2	COG3440:Predicted_restriction_endonuclease	PF13020.6:Domain_of_unknown_function_(DUF3883)
12	COG0645!!!COG1051	2	COG0645!!!COG1051:Predicted_kinase!!!ADP-ribose_pyrophosphatase_YjhB,_NUDIX_family	PF13238.6 PF13671.6
13	COG2746	2	COG2746:Aminoglycoside_N3'-acetyltransferase	PF02522.14:Aminoglycoside_3-N-acetyltransferase
14	COG4974	2	COG4974:Site-specific_recombinase_XerD	PF00589.22:Phage_integrase_family PF02899.17:Phage_integrase,_N-terminal_SAM-like_domain PF13495.6
15	COG0262	2	COG0262:Dihydrofolate_reductase	PF00186.19
16	COG1708	2	COG1708:Predicted_nucleotidyltransferase	PF13427.6:Domain_of_unknown_function_(DUF4111) PF01909.23:Nucleotidyltransferase_domain PF18765.1:Polymerase_beta,_Nucleotidyltransferase
17	COG2076	2	COG2076:Multidrug_transporter_EmrE_and_related_cation_transporters	PF00893.19:Small_Multidrug_Resistance_protein
18	COG2059	2	COG2059:Chromate_transport_protein_ChrA	PF02417.15:Chromate_transporter
19	COG1695	2	COG1695:DNA-binding_transcriptional_regulator,_PadR_family	PF13601.6:Winged_helix_DNA-binding_domain PF03551.14:Transcriptional_regulator_PadR-like_family PF02334.16:Replication_terminator_protein
20	COG2200	2	COG2200:EAL_domain,_c-di-GMP-specific_phosphodiesterase_class_I_(or_its_enzymatically_inactive_variant)	PF00563.20:EAL_domain
21	COG0425	2	COG0425:TusA-related_sulfurtransferase	PF02411.15:MerT_mercuric_transport_protein
22	COG3677	2	COG3677	PF12759.7:InsA_C-terminal_domain PF03811.13:InsA_N-terminal_domain
23	COG0640	2	COG0640:DNA-binding_transcriptional_regulator,_ArsR_family	PF12840.7 PF01022.20:Bacterial_regulatory_protein,_arsR_family
24	COG4747	2	COG4747:Uncharacterized_conserved_protein,_contains_tandem_ACT_domains	----
25	COG2329	2	COG2329:Heme-degrading_monooxygenase_HmoA_and_related_ABM_domain_proteins	PF03992.16:Antibiotic_biosynthesis_monooxygenase
26	COG0786	2	COG0786:Na+/glutamate_symporter	PF03616.14:Sodium/glutamate_symporter
27	COG0583	2	COG0583:DNA-binding_transcriptional_regulator,_LysR_family	PF00126.27:Bacterial_regulatory_helix-turn-helix_protein,_lysR_family
28	PF14082.6	2	----	PF14082.6:Domain_of_unknown_function_(DUF4263)
29	PF14319.6	2	----	PF14319.6:Transposase_zinc-binding_domain
30	PF05052.12	2	----	PF05052.12:MerE_protein
31	PF03203.14	2	----	PF03203.14:MerC_mercury_resistance_protein
32	PF13728.6	1	----	PF13728.6:F_plasmid_transfer_operon_protein
33	PF06122.11	1	----	PF06122.11:Conjugative_relaxosome_accessory_transposon_protein
34	PF07916.11	1	----	PF07916.11:TraG-like_protein,_N-terminal_region
35	PF18143.1	1	----	PF18143.1:HAD_domain_in_Swiss_Army_Knife_RNA_repair_proteins
36	COG0741	1	COG0741:Soluble_lytic_murein_transglycosylase_and_related_regulatory_proteins_(some_contain_LysM/invasin_domains)	----
37	COG1651	1	COG1651:Protein-disulfide_isomerase	PF10411.9:Disulfide_bond_isomerase_protein_N-terminus PF13098.6:Thioredoxin-like_domain PF01323.20:DSBA-like_thioredoxin_domain PF13462.6:Thioredoxin
38	COG0389	1	COG0389:Nucleotidyltransferase/DNA_polymerase_involved_in_DNA_repair	PF13438.6:Domain_of_unknown_function_(DUF4113) PF00817.20:impB/mucB/samB_family PF11799.8:impB/mucB/samB_family_C-terminal_domain
39	COG1974	1	COG1974:SOS-response_transcriptional_repressor_LexA_(RecA-mediated_autopeptidase)	PF00717.23:Peptidase_S24-like
40	COG4992	1	COG4992:Acetylornithine/succinyldiaminopimelate/putrescine_aminotransferase	----
41	COG2252	1	COG2252:Xanthine/uracil/vitamin_C_permease,_AzgA_family	PF00860.20:Permease_family
42	COG1142	1	COG1142:Fe-S-cluster-containing_hydrogenase_component_2	PF00037.27:4Fe-4S_binding_domain PF13237.6:4Fe-4S_dicluster_domain PF13247.6 PF13484.6:4Fe-4S_double_cluster_binding_domain PF12797.7 PF12798.7 PF12800.7 PF12837.7 PF12838.7 PF13187.6
43	COG0493!!!COG1142	1	COG0493!!!COG1142:NADPH-dependent_glutamate_synthase_beta_chain_or_related_oxidoreductase!!!Fe-S-cluster-containing_hydrogenase_component_2	PF01262.21:Alanine_dehydrogenase/PNT,_C-terminal_domain PF01494.19:FAD_binding_domain PF00037.27:4Fe-4S_binding_domain PF13247.6 PF14691.6:Dihydroprymidine_dehydrogenase_domain_II,_4Fe-4S_cluster PF12837.7 PF12838.7 PF13450.6:NAD(P)-binding_Rossmann-like_domain PF00070.27:Pyridine_nucleotide-disulphide_oxidoreductase PF07992.14 PF13738.6
44	COG2233	1	COG2233:Xanthine/uracil_permease	PF00860.20:Permease_family
45	PF12686.7	1	----	PF12686.7:Protein_of_unknown_function_(DUF3800)
46	COG1502	1	COG1502:Phosphatidylserine/phosphatidylglycerophosphate/cardiolipin_synthase_or_related_enzyme	PF13091.6:PLD-like_domain
47	COG1525	1	COG1525:Endonuclease_YncB,_thermonuclease_family	PF00565.17:Staphylococcal_nuclease_homologue
48	COG0270	1	COG0270:Site-specific_DNA-cytosine_methylase	PF00145.17:C-5_cytosine-specific_DNA_methylase PF18284.1:DNA_methylase_N-terminal_domain
49	PF06666.11	1	----	PF06666.11:Protein_of_unknown_function_(DUF1173)
