# Supplementary figures and images for: The Impact of Urban Pollution on Plasmid-Mediated Resistance Acquisition in Enterobacteria from a Tropical River
Source: Antibiotics (Basel). 2024 Nov 14;13(11):1089. doi: 10.3390/antibiotics13111089 (PMC11591392; doi:10.3390/antibiotics13111089)

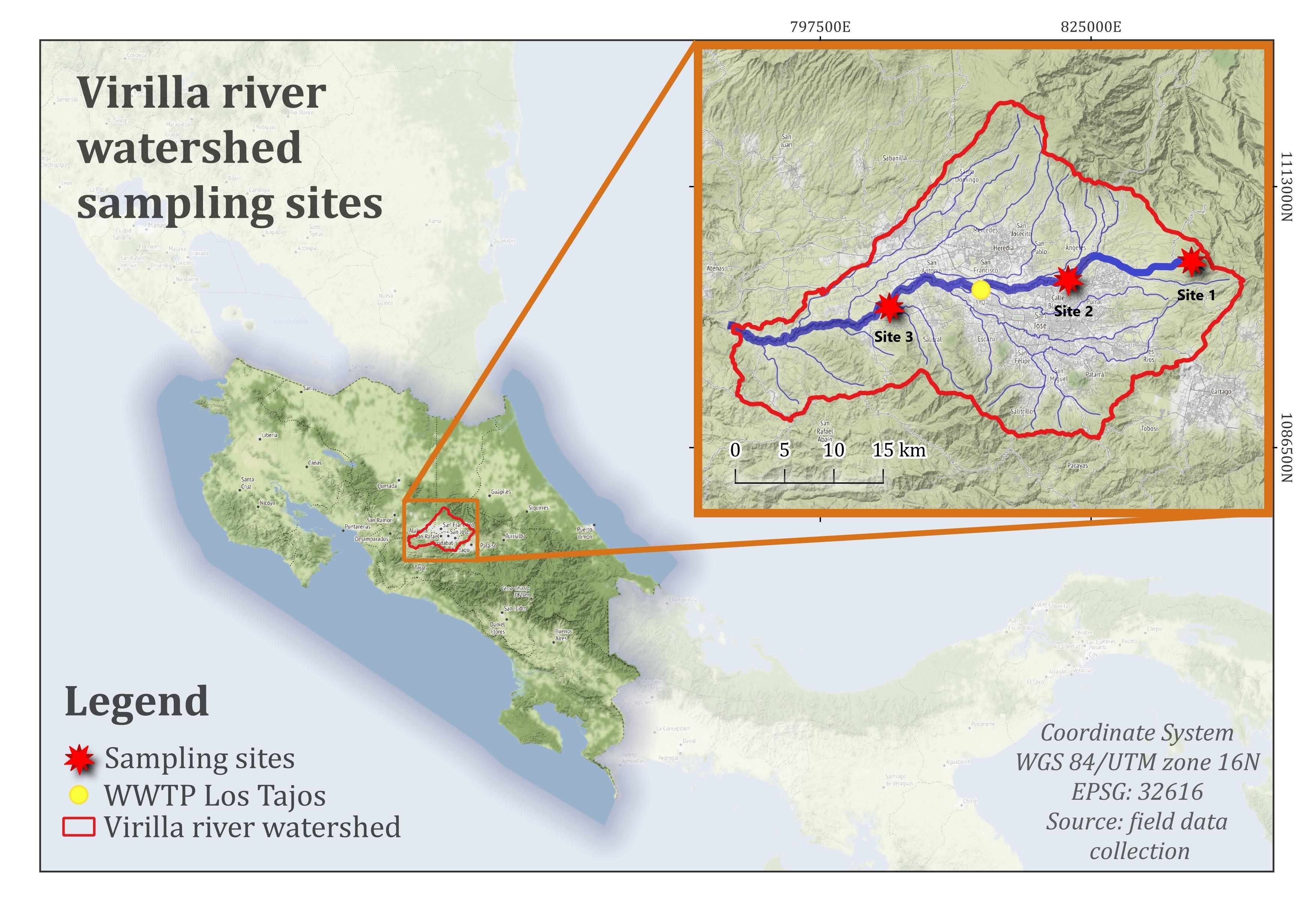

Supplement: Supplementary file 1 [file antibiotics-13-01089-s001.zip › Supplementary Figure S1.jpeg]

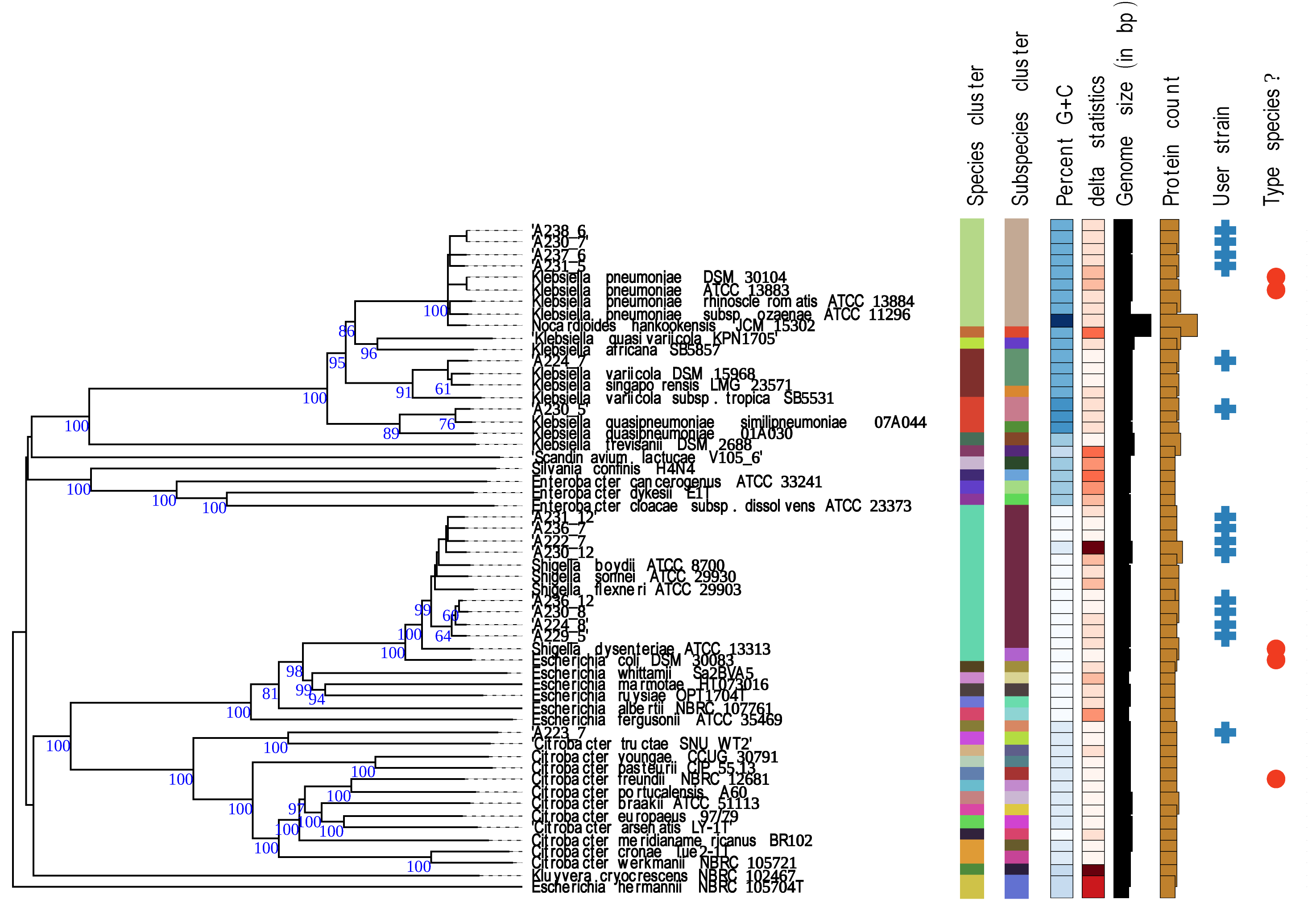

Supplement: Supplementary file 1 [file antibiotics-13-01089-s001.zip › Supplementary Figure S2.tif]
